# Comprehensive review on virtual reality for the treatment of violence: implications for youth with schizophrenia

**DOI:** 10.1038/s41537-019-0079-7

**Published:** 2019-07-23

**Authors:** Laura Dellazizzo, Stéphane Potvin, Sami Bahig, Alexandre Dumais

**Affiliations:** 1 Centre de recherche de l’Institut Universitaire en Santé Mentale de Montréal, Montreal, Canada; 2 Department of Psychiatry and Addictology, Faculty of Medicine, Université de Montréal, Montreal, Canada; 3 Institut national de psychiatrie légale Philippe-Pinel, Montreal, Canada

**Keywords:** Schizophrenia, Psychosis

## Abstract

Youth violence is a complex and multifactorial issue that has severe health and social consequences. While treatment options exist to treat/reduce violence in at-risk populations such as schizophrenia, there remains limitations in the efficacy of current interventions. Virtual reality (VR) appears to be a unique possibility to expose offenders and to train coping skills in virtual situations that are capable of eliciting aggression‐relevant behavior without threatening others. The focus of this paper is to provide a comprehensive review of studies using VR to manage violence across several at-risk populations, with a particular emphasis on youth with schizophrenia. Despite the encouraging success of VR applications for the treatment of different mental health problems, no studies have explored the usability of VR to specifically treat violence in patients with schizophrenia. A limited number of studies have focused on violence risk factors in other mental health problems (i.e., emotion regulation in individual suffering from post-traumatic disorders) that may be targeted in treatments to reduce the risk of violence. The preliminary studies using VR as a therapeutic element have shown reductions in anger, improvements in conflict-resolution skills as well as in empathy levels, and decreases in aggression. Possible applications of these interventions in youth with schizophrenia will be discussed.

## Introduction

Violence is a complex and multifactorial issue that has severe health and social consequences.^1^ The World Health Organization estimates that around 1.6 million deaths worldwide are due to violence, with 10–40 times as many physical injuries requiring medical attention.^1^ Youth violence in particular encompasses a myriad of behaviors ranging from homicide to lesser forms of aggressive behavior, such as bullying. It is a leading cause of death in young people and results in more than half a million medically treated physical injuries each year.^2^ The impact of youth violence goes well beyond physical consequences. Youth who experience violence as victims, perpetrators, or witnesses are at an augmented risk of physical health problems, engaging in other health risk behaviors, long-term emotional, behavioral, and mental health problems, as well as suicidal behaviors.^3–5^ Victims of violence are in their turn also at risk of perpetrating violence toward others.^6^

Violence often begins early in the life course of an individual, and many adolescents engaging in youth violence show patterns of disruptive behavior in early childhood.^7^ Adolescence and young adulthood are a time of heightened risk-taking behavior. Notably, there are diverse developmental trajectories that may lead to aggressive behaviors during adolescence and young adulthood.^8^ These pathways often begin with more mild behaviors (i.e., defiant behavior, delinquency) and progress to more severe behaviors later in life. While no sole risk factor alone leads to the development of violence, numerous factors associated with the future perpetration of violence are apparent early in the individual’s lifespan.^9^ These factors may be targeted in interventions to reduce the proclivity toward violence and include difficulties in emotion regulation (i.e., anger/hostility), self-regulatory symptoms (impulsivity), problem-resolution and social skills, as well as empathy.^10–13^

Markedly, emotional instability and behavioral impulsivity, among other problems, are also observed across numerous at-risk populations such as those with psychiatric disorders (i.e., post-traumatic stress disorder (PTSD), personality disorders, and severe mental illnesses), which are at an elevated risk of violence in comparison with the general population.^14,15^ More particularly related to severe mental disorders, such as schizophrenia, research asserts that these disorders are associated with an increased risk of violent and non-violent crime.^16^ It has also been shown that those with severe mental illnesses are at a greater risk of having multiple incarcerations compared with those without these disorders.^17–19^ While clear diagnoses of schizophrenia are infrequently given prior to early adulthood and are less common in juvenile justice settings,^20^ some youth may display psychotic-like symptoms that are possible expressions of an early form of a psychotic disorder.^21^ These symptoms may additionally lead to violent behavior.^22^ Different etiological subtypes of violence in psychosis have suggested that violent behaviors (i) are directly associated with positive psychotic symptoms, (ii) result from a comorbidity with personality disorders, and (iii) are triggered by impaired impulse control.^23^ Patients who exhibit antisocial behavior at a young age and continue to do so throughout their lifetime usually engage in violence prior to illness inception. Most acts of violence are committed by this antisocial subgroup. On the other hand, patients who show no sign of antisocial activity prior to illness and then commence an offending trajectory afterwards often exhibit a chronic course of schizophrenia, and display severe patterns of violence. In this latter subgroup, improving the symptomology of schizophrenia (i.e., reducing hallucinations and delusions) may reduce violence also, which is not necessarily the case for the former subgroup.^24^

Designing interventions to decrease the risk of violence in any psychiatric population is not an easy task and should address multiple risk factors. Some pharmacological interventions have been shown to have an effect in violence and hostility reduction.^25^ More prominently, in addition to being effective for treatment-resistant schizophrenia, clozapine has been regarded as the treatment of choice for persistent violence in comparison with other second-generation antipsychotics (i.e., olanzapine, risperidone).^26,27^ Other pharmacological treatments such as benzodiazepines and anticonvulsants have shown mitigated results.^28,29^ Furthermore, non-pharmacological interventions to manage violence are not widely available, and the results are not clearly conclusive.^30^ Some evidence exists for psychological interventions (i.e., cognitive-behavioral therapy (CBT)) for anger, aggression, and recidivism.^31,32^ Though, their effect sizes are at best moderate and it remains unclear whether their benefits would last in time and remain in higher-quality studies.^33^ Several restrictions to current interventions aiming to reduce aggression have been stated, such as limited exposure to provocations to practice anger management in real-life situations, limited social skills training, and difficulties in engaging at-risk populations in treatment.^34^ Furthermore, interventions for violence have been shortcoming as researchers/clinicians cannot ethically put individuals in a dangerous situation, even after providing them the skills to manage the corresponding situation, nor can they follow offenders in their daily lives while awaiting for the occurrence of risky situations to provide intervention skills.^35^

Virtual reality (VR) offers a possible remedy to such problems, as it has been shown that individuals tend to respond realistically to virtual simulations of real-life events.^36^ At the same time, the depicted situations are under the control of the clinician. Virtual environments have become a strong tool in mental health.^37,38^ In regards to violence, VR provides an instrument to study violent behaviors without exposing individuals to any true threat and thus overcomes the ethical issues that arise in non-VR projects.^39,40^ This has been previously shown in several studies in which VR was used to assess sexually deviant behaviors of child molesters, and to study bystander responses to a violent incident to better understand helping and aggressive behaviors.^36,41^ Particularly, VR seems to be a unique possibility to expose offenders and to train coping skills in virtual environments that are able to elicit aggression‐relevant behavior without jeopardizing others.^42^ Incorporating VR into treatment provides practitioners with more options leading to greater success with specific groups of offenders.^43^ Though, limited studies have explored the use of VR to manage and treat violence, and none to our knowledge has been conducted specifically in schizophrenia. For this reason, the primary scope of this article is to first review available literature regarding interventions using VR to manage factors associated with violence (i.e., emotion regulation, empathy) in any potential at-risk population. Since these studies are not conducted in a population diagnosed with schizophrenia, we will then discuss how the developed interventions targeting common difficulties may be adapted for the management of violence in youth with schizophrenia. The secondary scope is to further review VR therapies that are on risk factors of violence more specific to schizophrenia (i.e., hallucinations, persecutory delusions). We will also discuss their possible implications for reducing violence.

## Results

**Fig. 1:**
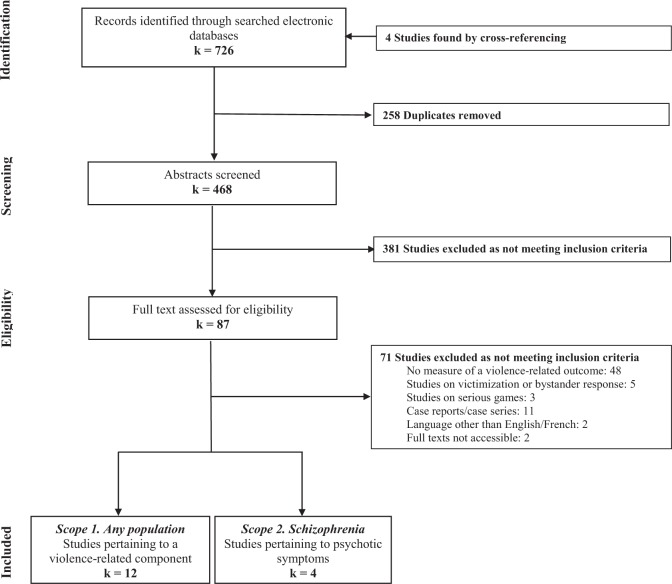
Flowchart depicting the search strategy employed to find the studies included in the review

**Fig. 2:**
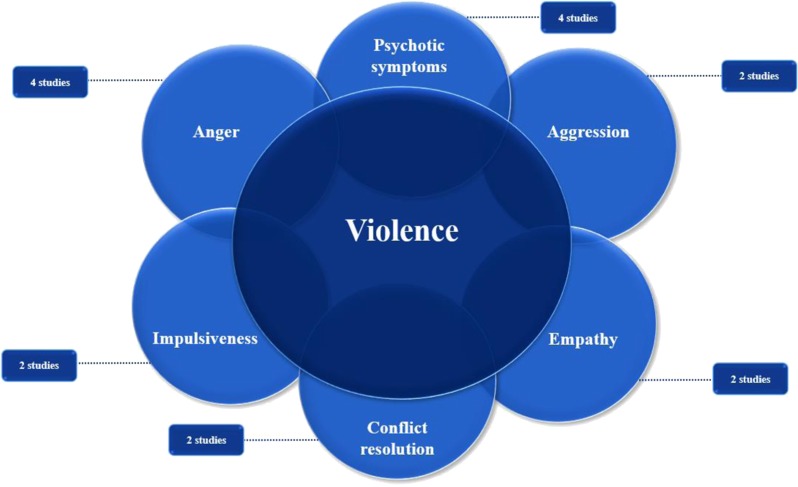
Summary of interventions assessing a violence-related component

As for our primary scope, 12 studies were extracted: four measuring anger in individuals with PTSD, two measuring impulsivity in juvenile offenders, two assessing conflict-resolution skills in adolescents and prisoners, two assessing empathy in violent offenders and middle school students and, lastly, two measuring aggression in veterans and forensic patients (see Tables 1–5). The latter study on forensic patients was a published protocol and did not present any results. As no studies were found using VR in schizophrenia or psychotic disorders to manage/treat violence, our secondary scope included VR interventions to treat psychotic symptoms related to violence. In all, four studies were extracted: one treating persecutory delusions and three treating auditory hallucinations (see Table 6). The PRISMA flowchart for the inclusion of studies in the review may be found in Fig. 1. See also Fig. 2 for a summary of the types of VR interventions assessing a violence-related component.

**Table 1:** Details of studies assessing anger

Author, year, geographic setting	Sample (*N*)	Age	Intervention				
			Conditions	Duration	Description	VR component	Main finding
Ba*ñ*os, 2011; Spain	PTSD, pathological, grief adjustment disorders (39)	18–50, M = 30.9, SD = 9.0	CBT; CBT+EMMA's World	Nine weekly sessions	CBT: education component, imagery exposure/restructuring the loss, relapse prevention.	Patients visualized a virtual environment that offered them a setting in which they could feel free to express their emotions. Their emotions would have an effect on the virtual world. The therapist had an important and active role in the customization of the environment during the session.	Significant ↓ in the frequency and intensity of anger
Difede, 2014; United States	PTSD following the WTC attack (25)	25–70, M = 45.8, SD = 10.5	VRE+DCS; VRE-placebo	12 weekly sessions	VRE: exposure.	Participants recounted their trauma in the first-person present tense, with as many sensory details as possible. A computer simulation of the September 11th (WTC) attacks was used to enhance an imaginal exposure therapy protocol.	Significant ↓ in anger
Beidel, 2017; United States	Veterans and active-duty military personnel with PTSD (112)	M=37.1, SD = 9.1	TMT	29 session in 3 weeks	TMT Intensive Outpatient Program: exposure therapy, programmed practice, social and emotional rehabilitation, social reintegration, anger management, brief behavioral activation.	Individual exposure therapy was conducted each morning and consisted of imaginal exposure augmented by virtual reality (Virtual Iraq/Afghanistan System).	Significant ↓ in anger
Beidel, 2017; United States	Veterans and active-duty military personnel with PTSD (92)	TMT: M = 37.7, SD = 8.5; VRET: M = 33.3, SD = 11.3	VRET+group TMT; VRET+group psychoeducation	29 session in 17 weeks	TMT: individual treatment first and consists of one psychoeducation/imaginal exposure therapy scene construction session, followed by 14 sessions of VRET, programmed practice, group treatment, social reintegration, anger management/problem solving, brief behavioral activation. VRET only: one education session, 14 VRET sessions, and 14 group treatment sessions.	Virtual Iraq/Afghanistan System consisted of a set of virtual environments for the treatment of combat-related PTSD.	Significant ↓ in anger

*CBT* cognitive-behavioral therapy, *DCS* D-cycloserine, *M* mean, *PTSD* post-traumatic stress disorder, *SD* standard deviation, *TMT* trauma management therapy, *VRE* virtual reality exposure, *VRET* virtual reality exposure therapy, *WTC* World Trade Center

**Table 2:** Details of studies assessing impulsiveness

Author, year, geographic setting	Sample (*N*)	Age	Intervention				
			Conditions	Duration	Description	VR component	Main finding
Cho, 2002; Korea	Juvenile offenders (50)	14–18	Desktop cognitive training; Desktop neurofeedback; VR cognitive training; VR neurofeedback; Control	Eight sessions in 2 weeks	Cognitive training: enhance focused and selective attention as well as sustained attention. EEG biofeedback: By controlling the EEG threshold levels, the virtual environment changed. As the score advances, a dinosaur egg rose from the desk. Then the egg was split into two pieces. From the broken egg, one part of a dinosaur picture appeared from the whiteboard gradually. Once all six parts of the picture were put together, the task was completed.	Virtual classroom: the immersive virtual classroom allowed youth to easily pay attention to the classroom environment. The small classroom had a whiteboard, a desk, a teacher avatar, a friend avatar, a large window looking out onto a playground, an entrance, several pictures hung on the wall, a sofa, a ceiling light, and a wooden floor. They could see themselves sitting at the desk. Training sessions were conducted in the virtual environment.	↓ in commission errors (not significant)
Cho, 2004; Korea	Juvenile offenders (28)	14–18	Desktop neurofeedback; VR neurofeedback; Control	Eight sessions in 2 weeks	EEG biofeedback.	Virtual classroom.	↓ in commission errors (not significant)

*EEG* electroencephalogram, *M* mean, *SD* standard deviation, *VR* virtual reality

**Table 3:** Details of studies assessing conflict-resolution and social skills

Author, year, geographic setting	Sample (*N*)	Age	Intervention				
			Conditions	Duration	Description	VR component	Main finding
Hubal, 2008; United States	African–American male adolescents in 10th grade, half had a diagnosis of conduct disorder (125)	M = 15.7	PACT+VR vignettes; No PACT+VR vignettes	/	PACT: violence prevention program developed for adolescents who are exposed to violence in their communities, families, and schools or who have exhibited a propensity for violent behavior, to improve their anger management and social-cognitive (i.e., negotiation and conflict-resolution) skills.	Virtual vignettes: simulated interpersonal verbal interactions with appropriate body language. Scripts were developed to induce students to engage in risky decision-making, and exhibit impulsive behavior.	PACT+VR group: significant ↑ in the use of positive interaction skills during the post-intervention vignettes. Improvement in negotiation and conflict-resolution skills
Hubal, 2008; United States	Prisoners (226)	21–49	CBT+VR vignettes	50 sessions CBT	CBT: helped patients recognize situations in which they are likely to become agitated or aggressive, avoid these situations when appropriate, and cope more effectively with a range of problems and behaviors associated with aggression.	Virtual vignettes: short, focused interactions used to examine dialogue, behaviors, and decisions made in real-world contexts. Each vignette invoked a specific cognitive function (risky decision-making, impulsivity, and sensitivity to penalties).	No differences between baseline and post-treatment outcomes

*CBT* cognitive-behavioral therapy, *M* mean, *PACT* positive adolescent choices training, *VR* virtual reality

**Table 4:** Details of studies assessing empathy

Author, year, geographic setting	Sample (*N*)	Age	Intervention				
			Conditions	Duration	Description	VR component	Main finding
Seinfeld, 2018; Spain	Offenders of domestic violence and control group with no history of violence (39)	21–61; Offender: M = 38.8, SD = 8.5; Control: M = 36.0, SD = 10.6	VR vignettes	/	VR vignettes: perspective-taking/role play.	The scenarios depicted a room with a long hallway where the offender’s own body was replaced with the body of a virtual female. The virtual body moved in real time in accordance with the actual movements of the participants. A male virtual character entered the room and began to verbally abuse the female virtual character following a pre-defined script.	Fear recognition difficulties and bias towards categorizing fear as happy improved
Ingram, 2019; United States	7th and 8th grade students (118)	11–14, M = 12.5, SD = 0.61	VR enhanced bullying prevention curriculum; Business-as-usual	Six sessions	VR enhanced bullying prevention curriculum: integrated the VR experience into standard practice of short-term bullying prevention.	The VR scenarios guided students through scripted adaptations of realistic bully relevant scenarios. Each focused on a different topic: standing up for victims, the consequences of common ineffective responses to bullying, and how to make a difference with small and realistic actions.	Significant ↑ in empathy levels

*M* mean, *SD* standard deviation, *VR* virtual reality

**Table 5:** Details of studies assessing aggression

Author, year, geographic setting	Sample (*N*)	Age	Intervention				
			Conditions	Duration	Description	VR component	Main finding
Zinzow, 2018; United States	Veterans with driving anxiety and aggression problems (8)	M = 36.5, SD = 8.4	VRET+CBT	Eight sessions	VRET+CBT: psychoeducation, relaxation skills training, training on the driving simulator, cognitive distortions introduction, exposure to driving scenarios, identifying anger triggers and cues, identifying values and how aggression interferes with them, review of coping skills, relapse prevention.	Nine scenarios for adaptation and training purposes. Military-based additions included loud noises; unexpected items beside the road; road construction; animals; abrupt movements by other vehicles; heavy traffic; left turns into oncoming traffic; being passed by other cars; being boxed in by other cars or impediments; tailgating vehicles; and various pedestrians, including those dressed in traditional Middle Eastern attire and/or holding objects that could resemble weapons.	↓ of PTSD symptoms, hyperarousal in driving situations, anxiety/anger-related thoughts/behavior and risky/aggressive driving
Tuente, 2018; Netherlands	Psychiatric forensic patients (128)	18–65	VRAPT; Treatment as usual	16-biweekly sessions	VRAPT: based on the SIP model.	Simulation that showed a virtual environment in which patients were confronted with behaviors and virtual characters in social situations.	Ongoing

*CBT* cognitive-behavioral therapy, *M* mean, *PTSD* post-traumatic stress disorder, *SD* standard deviation, *SIP* social information processing, *VR* virtual reality, *VRAPT* virtual reality, *VRET* virtual reality exposure therapy

**Table 6:** Virtual reality therapies for psychotic symptoms in schizophrenia

Author, year, geographic setting	Sample (*N*)	Age	Intervention				
			Conditions	Duration	Description	VR component	Main finding
Freeman, 2016; United Kingdom	Patients with persecutory delusions (30)	VR cognitive therapy: M = 42.1, SD = 13.4; VRE: M = 40.6, SD = 14.4	VR cognitive therapy; VRE	Within a day	VR cognitive therapy: threat belief tests in VR with the dropping of safety behaviors; the threat belief testing group; VRE: exposure; keeping of safety behaviors.	There were two VR environments: an underground train ride and an elevator. Each had gradations of difficulty based on the number of avatars placed around where the participant could walk.	Large ↓ in delusional conviction and real-life distress
Leff, 2013; United Kingdom	Patients with schizophrenia who hear persecutory voices (26)	14–75	AT; Treatment as usual	Seven weekly sessions	AT: computer technology that enabled the patient to create an avatar of the entity they believed was talking to them. The voice of the therapist was transformed in real time.	The therapist controlled the avatar so that it progressively came under the patients’ control. Over the course of the therapy the avatar was changed by the therapist from being abusive to being helpful and supportive of the patient.	Greater effects of AT on auditory verbal hallucinations
du Sert, 2018; Canada	Patients with schizophrenia/schizoaffective disorder who hear persecutory voices (19)	24–62, M = 42.9, SD = 12.4	AT; Treatment as usual	Seven weekly sessions	AT: patients created an avatar best resembling the most distressing person or entity believed to be the source of the malevolent voice, which was designed to closely have both the face and the voice of the “persecutor”. The avatar's voice was simulated in real time.	The immersive virtual environment consisted of an avatar standing in the dark, seen from a first-person perspective. Sessions 1 to 3, patients were confronted to the reproduced hallucinatory experience. Over the course of the therapy, the avatar's interaction with the patient became gradually less abusive and more supportive.	Greater effects of AT on auditory verbal hallucinations
Craig, 2018; United Kingdom	Patients with schizophrenia/affective disorders who hear persecutory voices (150)	18–65, M = 42.7, SD = 10.7	AT; Supportive counseling	Seven weekly sessions	AT: participants first created a computerized representation of the entity that they believed was the source of their main voice. A video link allowed the therapist to see and hear the participant’s responses, enabling them to adjust therapeutic interventions and modify the avatar interaction according to the unfolding dialogue.	Each session involved face-to-face work with the avatar, wherein the therapist facilitated a direct dialogue between the participant and the avatar. Phase one: exposure to the avatar speaking the typical verbatim content of the participant’s voices while the therapist encouraged assertive responding. Phase two: The dialogue gradually evolved as the avatar conceded ground and acknowledged the strengths and qualities of the participant.	Large effects of AT on distress associated with auditory verbal hallucinations

*AT* avatar therapy, *M* mean, *SD* standard deviation, *VR* virtual reality, *VRE* virtual reality exposure

### Emotion regulation: anger

By eliciting strong reactions, VR environments may facilitate the treatment of anger.^44^ VR stress-provoking scenarios may enable individuals to develop self-regulatory skills and enhance exposure treatments to manage the expression of uninhibited anger.^45^ Four studies assessing anger as a target symptom of stress-related disorders were carried (see Table 1). These studies are interesting since PTSD patients may respond aggressively to perceived threats, which may be similar to patients with schizophrenia developing persecutory delusions and acting violently on their beliefs.

To begin, Baños et al.^46^ conducted a controlled study comparing nine-weekly sessions of standard CBT to a CBT program with “EMMA’s World” in 39 patients with PTSD, Pathological Grief and Adjustment Disorders. EMMA’s World VR-system showed customized trauma-related environments that used symbols and tailored aspects to encourage emotional responses in patients. The system was, moreover, manipulated by the therapist. Such responses may in turn help patients to emotionally process negative events in a harmless setting. The results proceeding both interventions showed a significant decrease in the frequency and intensity of anger. Subsequently, Difede et al.^47^ conducted a pilot randomized trial on a 12-week VR exposure therapy in 25 patients with chronic PTSD following the World Trade Center (WTC) attacks. A computer simulation of the WTC attacks was used to enhance imaginal exposure therapy. In this context, many patients may prefer to avoid reminders of their traumatic event, and are reluctant/incapable of visualizing effectively the distressing event. VR creates safe and controllable situations that may be more tolerable to individuals than in vivo exposure.^48^ The patients were randomized to obtain either a cognitive enhancer or a placebo before exposure. Both groups showed significant reductions in their PTSD symptoms including anger compared with the baseline, suggesting that VR exposure therapy may hold a promise for the treatment of chronic PTSD. Lastly, Beidel’s team used a Trauma Management Therapy (TMT) by adding VR to the exposure therapy component. First, their controlled pilot study evaluated the efficacy of a 3-week intensive outpatient program for combat-related PTSD in 112 veterans and active-duty personnel.^49^ Second, they conducted a randomized controlled study comparing the efficacy of two 17-week interventions in veterans and active-duty personnel as well: (i) TMT combined with VR exposure therapy and (ii) VR exposure therapy combined with psychoeducation.^50^ The first phase of both treatment groups was VR exposure therapy. Both their studies indicated statistically significant decreases of core PTSD symptoms, as well as significant decreases in anger. Particularly, the reduction in anger occurred after the VR exposure component of the intervention. Treatment gains were sustained at the 6-month follow-up. Overall, the preliminary results of these interventions for PTSD symptoms using a VR exposure component have shown promising results in anger management.

### Impulsiveness

Impulsivity, which is seen in a number of psychiatric disorders including schizophrenia, has been shown to lead to problematic behaviors, such as violence.^51^ The use of VR in cognitive training, mainly for the assessment of attention difficulties and rehabilitation, has been shown to be an alternative tool.^52^ Cho’s team developed a cognitive training program with immersive VR based on a virtual classroom and electroencephalogram (EEG) biofeedback for symptoms of attention-deficit hyperactivity disorder (ADHD)^53,54^ (Table 2). The virtual classroom has been used previously to enable individuals to solve tasks, while different stimuli were presented.^52^

Their first clinical trial comprised 50 participants aged between 14 and 18 who had committed crimes in the past and who were in a youth detention center.^53^ While the adolescents did not have a formal diagnosis of ADHD, they did show signs of learning difficulties (i.e., inattentive, impulsive, hyperactive, and distracted). They were assigned to either the (i) control group, (ii) desktop cognitive training group, (iii) desktop neurofeedback group, (iv) VR cognitive training group, or (v) VR neurofeedback group. The non-VR and VR groups underwent eight sessions over two weeks, while the control group had no training session. Cognitive training groups were meant to enhance focused and selective attention, as well as sustained attention. The individuals in the neurofeedback training groups were connected to an EEG acquisition device. Once the EEG signal was greater than a targeted threshold level, the virtual environment would change as a positive reinforcement (a part of a puzzle in the virtual environment would be filled in until its completion). All participants performed a continuous performance task (CPT) before and after the complete training sessions. Commission errors were assumed to represent impulsive behavior. Similarly, their second study randomized 28 male juvenile offenders.^54^ The three groups consisted of a (i) control group (no training session), (ii) neurofeedback with VR group, or (iii) neurofeedback only group (with a computer monitor). The VR and non-VR groups both underwent the same neurofeedback training tasks with the exception that the VR group could look around in the VR classroom. After training, both the VR and non-VR groups showed fewer commission errors. Although not significant, the VR group improved the most. This highlights that immersive VR may be added to neurofeedback to treat impulsivity.

### Conflict resolution and social skills

Studies have also identified socio-cognitive predictors as risk factors for violence in schizophrenia such as poor conflict resolution, poor facial emotion recognition, or reduced ability to discriminate intensities of facial emotions.^55,56^ VR is a good way to allow at-risk individuals practice vital social skills (i.e., emotional control, expressing one's preferences, negotiating, compromising, and using non-provocative language).^35^ Hubal et al.^35^ conducted two studies using embodied conversational agents (ECA) and hypothetical social situations (“virtual vignettes”) to help develop social competency skills in different at-risk populations (Table 3).

Their first study included 125 African–American male adolescents with half having a diagnosis of a conduct disorder. Whereas the experimental group received the Positive Adolescent Choices Training (PACT) including virtual vignettes, the other half did not receive the PACT intervention. The PACT prevention program aimed to improve anger management and social-cognitive skills. Vignettes were created to simulate provocative interpersonal social situations in a school setting. The authors noted that the participating adolescents were sufficiently engaged in the vignettes that they conducted risk-taking behavior even after exposure to the preventive materials. Those who received the PACT program were significantly more likely to use positive interaction skills during the post-intervention vignettes than those who were not exposed. Their results indicate some efficacy for the PACT intervention, as well as for the use of VR vignettes. Findings also suggest that the PACT program may improve negotiation and conflict-resolution skills in adolescents who are at an elevated risk of becoming perpetrators and/or victims of violent behavior.

Their second study comprised a CBT program with a succession of ECA virtual vignettes in a sample of 226 adult male offenders. The CBT program aimed to aid offenders recognize situations in which they were likely to become aggressive, avoid such situations when suitable, and better cope with problems and behaviors related to aggression. The vignettes consisted of short, focused interactions to examine their discourse, behaviors, and decisions made in a real-world context. However, in comparison with their first study, there were no differences between baseline and post-treatment outcomes. They stipulated that this may have been due to the prisoners’ unfamiliarity with the technology, as well as their unwillingness to express their responses being that they are more closely monitored in prison settings. Furthermore, this difference may have been due to the pre-defined fixed vignettes that were not personalized to the offenders’ specific needs.

### Empathy

The perpetration of violent acts against others has been linked to a lack of empathy and to a deficit in the ability of offenders to put themselves in the standpoint of victims. Studies have also found that offenders have difficulties in accurately recognizing emotions (i.e., fear and anger) as stated above, which have been hypothesized to hinder offenders’ empathetic responses.^57^ Providing individuals with empathy training may therefore help reduce aggressive behavior as well as hostile responses, while increasing the inclination to conduct socially appropriate behavior.^58,59^ Two studies have used VR scenarios to target empathy, both as a preventative measure and as skills-training (Table 4). In all, these studies further show that VR may have the potential to reduce the occurrence of violence by enabling both youth and adults to take on the role/perspective of victims of violent acts.

Ingram et al.^60^ designed VR scenarios that were meant to place adolescents in real-life situations, such as a party or in the hallway observing a fight. These scenarios were included in their program to prevent bullying. They therefore conducted a randomized pilot trial in 118 students to evaluate the effects of their VR enhanced bullying prevention program compared with business-as-usual. The study sample included any student and not solely students who had bullied others. Their results showed a significant increase in empathy levels after the VR intervention. Moreover, the latter intervention was associated with a reduction in the perpetration of bullying, which was mediated by empathy.

Seinfeld et al.^61^ used immersive VR to allow male offenders to experience virtual violent situations from the first-hand perspective of victims of domestic violence. Their study compared offenders to controls with no history of violence and found that VR perspective-taking enhanced emotion recognition in violent individuals. Hence, the findings showed that once offenders were put in the place of a female victim through VR, their fear-recognition difficulties in female faces as well as their bias toward categorizing fear as happy improved. This study highlights that offenders may augment their emotion recognition skills by allowing them to change their perception through immersive VR, which may have an impact on violence reduction.

### Aggression

Fairly few VR interventions have targeted aggression as a primary outcome. Two studies have developed interventions that have a direct impact on reducing aggressive behaviors in at-risk populations (Table 5). Such VR therapies should allow patients to learn several key strategies to control their aggression that may be applied later in real-life situations.

Zinzow et al.^62^ used a driving simulator to place military populations with aggressive driving problems in personalized VR-driving scenarios. They led a pilot study on eight veterans using their eight-session intervention combining VR exposure to CBT. The latter integrated anxiety and anger management as well. During each VR exposure, the veterans had the possibility to practice CBT skills that they had learnt. Findings showed notable changes on measures of driving aggression and anger. The authors thus found significant declines of PTSD symptoms, hyperarousal in driving situations, anxiety/anger-related thoughts and behaviors, and risky/aggressive driving, which lasted up to the follow-up.

Tuente et al.^63^ designed Virtual Reality Aggression Prevention Training (VRAPT), which is a treatment for reactive aggression in forensic populations. For the moment their research protocol has been published. The main goal of their ongoing randomized controlled trial is to evaluate the efficacy of their 16-biweekly individual training sessions using VR environments to reduce aggressive behaviors while comparing it to a waiting list in 128 forensic psychiatric inpatients. The VR environments place individuals in quarrelsome social situations with virtual individuals that are entirely controlled by an instructor (i.e., speech, emotional expressions, and body movements). Patients may thus learn to control their aggressivity and practice their skills at their own pace in a safe environment. VRAPT is another intervention that may be personalized to each person’s own objectives and difficulties. For the moment, they have stipulated that VRAPT will decrease aggressive behavior up to the 12-week follow-up as reported by patients and staff personnel. Other outcomes of interest also related to violence (i.e., anger and impulsivity) will be measured in their project. While the results of their study are not available yet, the intervention appears propitious for the treatment of violence more specifically. This intervention will certainly be of use for other populations and may be used as much as for preventative measures as for the management of violence.

### Virtual reality therapies for schizophrenia: potential impact on violence

Whereas research strongly supports the association between the occurrence of violence and psychosis, there are studies suggesting that the relation between symptomatology, violence and schizophrenia is not random, but directed by specific patterns of psychotic symptoms mainly belonging to positive symptoms.^16^ In terms of positive symptoms, command hallucinations, paranoid delusions, persecutory hallucinations have all been shown to raise the risk of violent behavior toward others. These symptoms have been associated with a threefold increase in the odds of violence.^64^ Literature also suggests that feelings of distress in relation to these symptoms may predict violence.^65,66^ The content of the symptoms is likewise relevant. For instance, delusions of being spied on, persecution as well as beliefs that one has been replaced by an imposter have been associated with violence. This is predominantly the case when these delusional beliefs occur with suspiciousness, hostility, and agitation.^67–69^ In addition, persecutory auditory verbal hallucinations are known to be distressing, as they often criticize the voice hearer, and occur with a significant frequency during extended periods of time.^70^ It has been suggested that command hallucination ordering individuals to conduct acts of violence toward others may increase their compliance and thus favor violent behavior.^71^ VR therapies have thus been developed to help patients cope with their distress and treat the presence of these psychotic symptoms.^72^

Freeman et al.^73^ anticipated to establish the potential therapeutic use of VR for delusions (Table 6). They developed an intervention for patients with persecutory delusion and conducted a proof-of-concept study, in which 30 patients were randomized to VR cognitive therapy or VR exposure. The virtual environments consisted of an underground train and an elevator. Each had gradations of difficulty based on the number of avatars placed around the patient. In comparison with exposure alone, the VR cognitive therapy led to large reductions in delusional conviction and real-life distress.

Avatar Therapy (AT) has been a new advancement for the treatment of auditory hallucinations. AT allows patients to create a visual representation (i.e., an avatar) of their persecutor. Patients are then encouraged to engage in a dialogue with their avatar animated entirely by the therapist. The therapeutic objective is to help patients gain control over their symptoms through emotion regulation. The results of the pilot trials of Leff et al.^74^ as well as du Sert et al.^75^ comparing AT with treatment as usual showed greater effects of AT on auditory verbal hallucinations. Craig et al.^76^ further conducted a larger single-blind randomized controlled trial comparing seven-weekly sessions of AT to supportive counseling in 150 patients with distressing auditory verbal hallucinations. The results showed large effects of the therapy on distress associated with auditory verbal hallucinations (Cohen's d = 0.8) compared with supportive counseling.

While none of these VR interventions have measured a violent outcome, it may be hypothesized that treating these crucial risk factors may have an impact on managing and preventing violence. Future well-controlled studies in schizophrenia are necessary in this area that will measure both violence and psychotic symptoms.

## Discussion

This review aimed to investigate the current state of knowledge regarding the treatment of violence using VR. A limited number of VR paradigms exists to treat at-risk individuals. The preliminary studies in populations other than schizophrenia have shown reductions in anger and impulsivity, improvements in conflict-resolution skills as well as in empathy levels and decreases in aggression. Particularly related to VR interventions for schizophrenia, reductions in delusions and auditory hallucinations were found, though they were not related to violence since this outcome was not measured.

More particularly, we were interested in determining the possible application of VR interventions for violence management in youth with schizophrenia. Since those with schizophrenia are affected by a panoply of symptoms, we investigated other psychiatric disorders that share common symptomatology, and especially transdiagnostic symptoms such as difficulties regulating strong negative affect, lack of necessary skills to resolve problems, and lack of empathy toward others. VR therapies tackling these important risk factors of violence may be included in future therapies for violent youth with psychosis. Moreover, although many of these VR interventions use fixed scenarios, EMMA’s World^46^ and VRAPT^63^ highlight the future of personalized therapies for violence management. Above the known risk factors of violence that may be targeted in at-risk populations, schizophrenia is also particularly affected by specific risk factors, such as the presence of psychotic symptoms.^16,17^ For the moment, symptom-specific VR therapies in schizophrenia for persecutory delusions and auditory verbal hallucinations exist.^73–76^ It may be hypothesized that treating these psychotic symptoms may reduce violence. Further studies are warranted in this area to elucidate if reducing psychotic symptoms will result in a reduction of violence.

Tailored novel VR approaches that go beyond the *one size fits all* approach are therefore needed to reduce violent behaviors in at-risk individuals, while reducing psychotic symptomatology and other transdiagnostic symptoms (i.e., anger). Such a holistic approach will be able to be more inclusive of the difficulties that must be treated in violent youth with schizophrenia. In all, current literature suggests that an immersive VR therapy combining elements of AT^74–76^ for hallucinatory symptom reduction with exposition to at-risk situations that may trigger perceived threat, as well as allow skill training^46,47,49,50,62,63^ used to manage negative emotions, impulsivity, and violence should be the next few steps. More specifically, such VR therapies will enable young offenders to learn and practice relevant skills in real time with the therapist to reduce violence. Moreover, it has been proposed that using more immersive VR systems, such as high-quality head-mounted display (HMD) instead of a lesser immersive system (i.e., computer monitor), will lead to higher levels of presence and more emotional arousal, which are necessary components for better treatment efficacy.^77^

Due to the preliminary stages of VR as a treatment for violence, studies show numerous methodological shortcomings, including small sample sizes, lack of follow-up periods, and lack of consideration of cofounding factors.^72^ For instance, individuals on psychotropics while being treated with VR could be an important confounding factor. Indeed, patients with schizophrenia or other psychiatric disorders such as PTSD are often on psychotropics, which could have an effect on the intervention if not controlled for in the study. For the studies that have specified that participants were on psychotropic medication,^47,49,50,74–76^ they had to be on stable medication throughout the intervention, it is thus unlikely that medication impacted the results. Furthermore, these results should be taken carefully as studies all evaluated a different type of intervention with a VR component, and the VR component was not necessarily the principal part of the intervention. In addition, apart from the protocol of Tuente et al.^63^ aiming to assess several violence-related measures, studies assessed one type of factors (i.e., only anger, only impulsivity). Though, these measures are not independent from one another; for instance, managing anger may result in a reduction of impulsivity. Future studies should include more violence outcome measures and especially risk-assessment scales (i.e., HCR-20^78^). The processes involved in VR therapies remain relatively unexplored, and studies have not generally verified how the findings translate to real-life situations. Finally, it is not possible to determine if interventions in other at-risk populations (e.g., PTSD) can indeed be applied to patients with a diagnosis of schizophrenia, even though common and transdiagnostic risk factors, such as emotion regulation, are targeted. Future studies adapting the essence of these VR interventions in schizophrenia should be carried out to verify the efficacy of specific and non-specific interventions to reduce violence.

There is a clear need to implement better prevention programs and management targeting violence amid at-risk populations; one such approach may be with the inclusion of VR. Given the positive results from intervention research in other populations, this review shows that VR may also be used for the management of violence risk in youth with schizophrenia. Future studies in young people with schizophrenia are warranted to evaluate the efficacy of VR therapies for violence.

## Methods

### Search strategy

A search was conducted on the electronic databases of PubMed, PsycINFO, and Google Scholar from the year 1990 (when VR began to be used in mental health) using search terms chosen to be inclusive for virtual reality (e.g., “virtual”, “virtual reality”, “VR”), violent behaviors or related constructs (e.g., “violence”, “aggression”, “anger”), and interventions (e.g., “intervention”, “therapy”). Reference lists were scanned by hand to identify additional studies. Searches were completed by January 2019. Abstracts and full texts were screened by L.D. and S.B. No setting, date, or geographical restrictions were applied; searches were limited to English or French language sources.

### Study eligibility

We did not restrict the search to any specific psychiatric population or any particular age group to achieve a maximum number of studies. Studies were included so long as they (1) enrolled any participants who received an intervention that comprised any type of VR component; (2) included a measure on a violent-related outcome (e.g., anger, impulsivity, aggression, conflict resolution, empathy). This allowed in the inclusion of the most studies on the subject. As for our secondary scope, we searched literature for specific VR therapies for schizophrenia that targeted risk factors of violence such as psychotic symptoms (i.e., hallucinations, delusions). Studies were excluded if they (1) were only on victimization or bystander behaviors; (2) used serious games; (3) were case reports/case series. Full texts that were not accessible were excluded as well.

### Data extraction

The data were extracted with a standardized form. Key information related to the sample and the intervention were recorded. The details of the studies may be found throughout Tables 1–6. Extracted data were independently cross-checked and any queries were resolved by discussion with A.D. and S.P. Furthermore, L.D. and S.P. independently undertook quality assessment using a set of criteria based on the GRADE Checklist.^79^ Studies were assigned: high, moderate, low, and very low quality (see Supplementary Material). To achieve a high standard of reporting data, the Preferred Reporting Items for Systematic Reviews and Meta-Analyses (PRISMA) guidelines was followed.^80^

## Supplementary information

Table S1

## Data Availability

No data sets were generated or analyzed for this study.

## Supplementary information

**Supplementary information** accompanies the paper on the *npj Schizophrenia* website (10.1038/s41537-019-0079-7).
