# Supplementary material for: Comprehensive review on virtual reality for the treatment of violence: implications for youth with schizophrenia
Source: NPJ Schizophr. 2019 Jul 23;5:11. doi: 10.1038/s41537-019-0079-7 (PMC6650426; doi:10.1038/s41537-019-0079-7)
Supplement: Supplementary file 1 — Table S1 [file 41537_2019_79_MOESM1_ESM.pdf]

Supplementary table 1. Quality assessment of the studies included in the comprehensive review based on the GRADE checklist

Study type	Author, year	Quality assessment based on the GRADE checklist
Randomized studies	Banos, 2011	High
	Difede, 2014	Moderate
	Beidel, 2017	Moderate
	Beidel, 2017	High
	Cho, 2002	Very low
	Cho, 2004	Very low
	Ingram, 2019	Moderate
	Tuente, 2018	High
	Freeman, 2016	Moderate
	Leff, 2013	Moderate
	du Sert, 2018	Low
	Craig, 2018	High
Observational studies	Hubal, 2008	Moderate
	Hubal, 2008	Moderate
	Seinfeld, 2018	Low
	Zinzow, 2018	Moderate
